# Reciprocal Asymmetric Transmission in Directionally Metallized Silica Nanogratings With Broken Lateral Mirror Symmetry

**DOI:** 10.1002/nap2.70244

**Published:** 2026-08-08

**Authors:** Ye Ming Qing, Yi Shen, Shunsuke Murai, Katsuhisa Tanaka, Koichi Okamoto

**Affiliations:** ^1^ Department of Physics and Electronics Osaka Metropolitan University Osaka Japan; ^2^ College of Electronic and Optical Engineering & College of Flexible Electronics (Future Technology) Nanjing University of Posts and Telecommunications Nanjing China; ^3^ Department of Material Chemistry Kyoto University Kyoto Japan

**Keywords:** broken lateral mirror symmetry, diffraction grating, lateral asymmetric transmission, reciprocal photonics

## Abstract

We study here lateral asymmetric transmission (AT), a phenomenon in which the transmission under illumination from the same half‐space depends on the sign of the in‐plane wavevector, $+k_{x}$ versus $-k_{x}$. Such lateral AT can emerge in reciprocal systems without polarization conversion when broken lateral mirror symmetry is combined with multiport diffraction. We experimentally and numerically investigate this effect in directionally Ag‐coated ${\text{SiO}}_{2}$ nanogratings, whose one‐sided metal coating breaks the lateral mirror symmetry. Rigorous coupled‐wave analysis shows that channel opening establishes the multiport scattering basis, whereas structure‐dependent resonant responses modulate the redistribution of power among transmission, reflection, and absorption channels. Field and normalized local‐loss distributions corroborate stronger metallic dissipation under $+k_{x}$ excitation and greater transmission under $-k_{x}$ excitation. Angle‐resolved measurements and morphology‐informed simulations show the same sign and principal angular–spectral evolution of the lateral transmission contrast. The design principle is further examined in directionally Al‐coated PMMA–${\text{TiO}}_{2}$ gratings on a ${\text{SiO}}_{2}$ substrate. These results identify broken lateral mirror symmetry as the structural prerequisite and higher‐order diffraction‐channel opening as the multiport basis for reciprocal lateral AT, with structure‐dependent resonant coupling modulating the attainable contrast.

## Graphical abstract

Directional silver coating breaks lateral mirror symmetry in a reciprocal silica nanograting. Opposite in‐plane illumination directions redistribute optical power differently among diffraction and absorption channels, producing strong polarization‐preserving lateral transmission contrast.

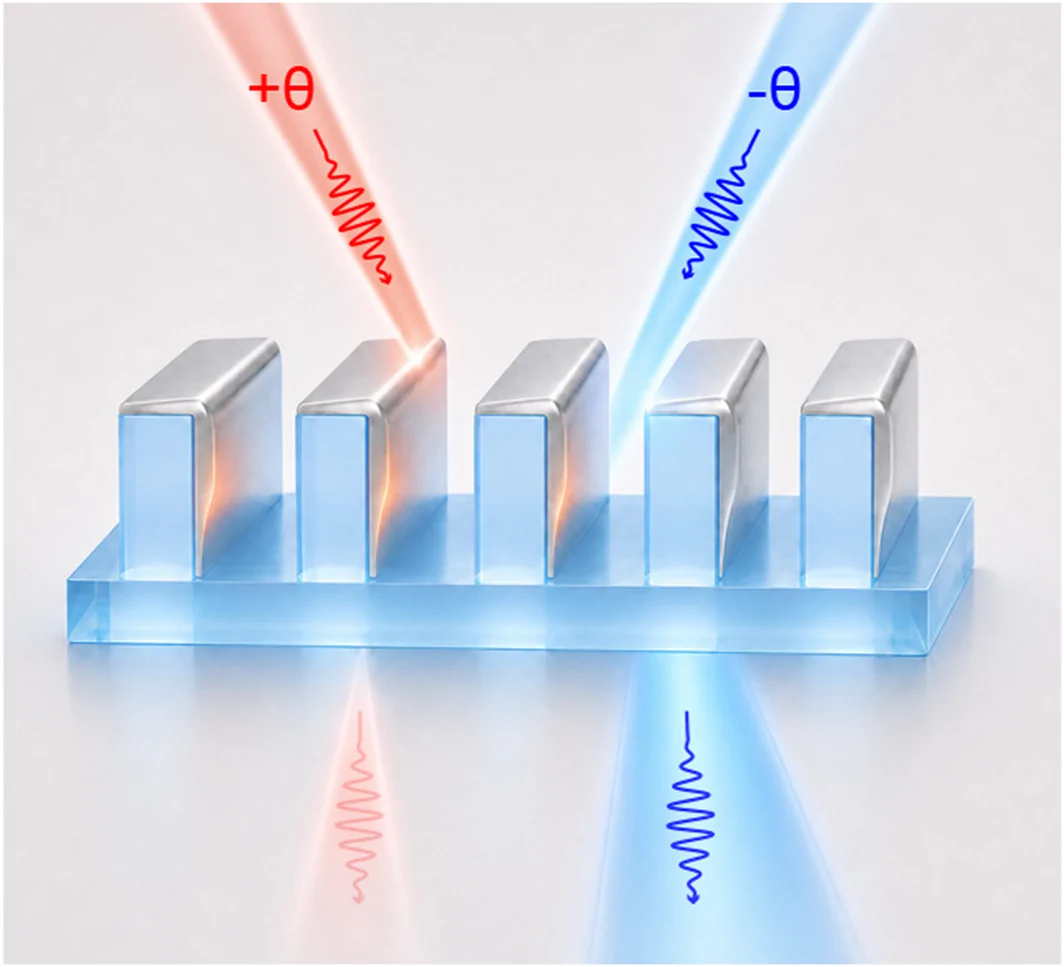

## 1 Introduction

Asymmetric transmission (AT) provides a route to direction‐selective optical functionality in reciprocal photonic systems and has therefore attracted sustained interest in nanophotonics. Within reciprocal AT, two excitation geometries should be distinguished. Vertical AT compares illumination from opposite half‐spaces of a structure, whereas lateral AT compares opposite in‐plane wavevectors, $+k_{x}$ and $-k_{x}$, under illumination of the same fixed structure from the same half‐space. In contrast to nonreciprocal transport, which requires breaking time‐reversal symmetry through magnetic bias, temporal modulation, or optical nonlinearity, reciprocal AT arises in linear passive structures when opposite illumination directions couple differently to the available optical channels [1, 2, 3, 4, 5, 6, 7, 8, 9, 10, 11]. This distinction is essential because reciprocal AT can offer compact and fabrication‐compatible alternatives for directional light management without invoking nonreciprocal materials or dynamic biasing [12, 13, 14].

Early studies of reciprocal AT were mainly developed in planar chiral and multilayer structures, where forward and backward illumination commonly referred to excitation through the opposite faces of a device and was often accompanied by polarization conversion [15, 16, 17]. These configurations are referred to here as vertical AT. By contrast, a smaller body of diffraction‐mediated studies has considered opposite in‐plane wavevectors under illumination from the same half‐space [18, 19]. We refer to these configurations as lateral or angularly asymmetric responses, according to the excitation geometry and terminology used in the respective studies. Distinguishing these two geometries is essential because they compare different pairs of incident channels, even though both remain subject to Lorentz reciprocity. More recently, theoretical studies have established that polarization‐preserving vertical AT in reciprocal laterally symmetric gratings can be enabled by diffraction‐order engineering and the associated multiport scattering response [12]. Related diffraction‐mediated implementations of vertical AT have also been reported [20, 21]. For a fixed polarization, a grating supporting only one propagating reflection channel and one propagating transmission channel behaves as an effectively two‐port system, for which reciprocity enforces equal transmission between reciprocal channels. Once one or more higher‐order diffraction channels become propagating, additional scattering ports are opened. Reciprocity continues to relate each diffraction channel to its reciprocal counterpart, but it does not generally require the total transmitted powers, summed over all propagating transmitted diffraction orders, to be identical for illumination from the top and bottom half‐spaces of the grating structure. Higher‐order diffraction therefore provides the multiport channel‐space prerequisite for polarization‐preserving vertical AT. If the grating additionally lacks lateral mirror symmetry, its total transmission under illumination from the same half‐space can also become laterally asymmetric, yielding different responses for the $+k_{x}$ and $-k_{x}$ lateral excitation configurations, as, for example, reported in refs. [18, 19]. The opening of an additional diffraction channel does not by itself guarantee a strong transmission contrast; the magnitude and spectral evolution of the contrast are subsequently determined by the broken lateral mirror symmetry and the structure‐specific redistribution among the open diffraction channels and material dissipation. Among these routes, plasmonic and hybrid metal–dielectric gratings are particularly attractive because they naturally combine diffraction, cavity confinement, and metal‐assisted surface waves within compact geometries [22, 23, 24, 25]. Within this multiport framework, structural resonant responses determine how power is redistributed among the open diffraction channels rather than providing an independent origin of polarization‐preserving AT. In metallic gratings, SPP‐related, localized‐plasmonic, and hybrid metal–dielectric responses can modify the coupling to the propagating diffraction orders and enhance the accompanying Ohmic dissipation. In dielectric gratings, localized or guided resonances can instead reshape the radiative coupling among the open diffraction orders without introducing metallic absorption. The opening or cutoff of each diffraction order is identified by its medium‐specific Rayleigh–Wood anomaly (RWA), which marks a kinematic channel threshold rather than an independent localized or plasmonic resonance. Accordingly, SPP‐related asymmetric responses in reciprocal gratings should be understood as resonance‐assisted power redistribution within a multiport diffraction landscape rather than as effects caused by SPP excitation alone [12, 26, 27, 28]. However, in simple reciprocal plasmonic–dielectric gratings operating in the visible and near‐infrared, a physically transparent picture linking branch‐resolved spectral asymmetry, field localization, absorption asymmetry, and realistic fabrication‐induced deformation is still comparatively limited. For polarization‐preserving AT in reciprocal gratings, propagating higher‐order diffraction channels provide the multiport condition required for unequal total transmission between reciprocal excitation configurations, irrespective of whether the relevant structural mirror symmetry is broken laterally or along the surface‐normal direction [12, 19]. Reflection and absorption differences should therefore not be regarded as alternative fundamental origins of the effect. Instead, they quantify how the direction‐dependent redistribution among the propagating diffraction channels and material dissipation is manifested in the total energy balance. For the present laterally asymmetric grating, the relevant question is therefore how the multiport redistribution is partitioned among reflection, transmission, and metallic absorption.

Here, we experimentally and numerically investigate polarization‐preserving reciprocal lateral AT in directionally Ag‐coated ${\text{SiO}}_{2}$ nanogratings with broken lateral mirror symmetry. By combining angle‐resolved transmission measurements with morphology‐informed rigorous coupled‐wave analysis, we distinguish the respective roles of structural symmetry breaking, higher‐order diffraction, and structure‐dependent field localization and dissipation. The one‐sided Ag morphology allows the reciprocal $+k_{x}$ and $-k_{x}$ excitation configurations to couple differently to the structure, whereas the opening of higher‐order propagating diffraction channels establishes the multiport scattering environment required for unequal total transmission. The order‐resolved analysis further reveals an evolution from predominantly reflection‐compensated transmission contrast at 500 nm, through comparable reflection and absorption contributions near the air‐side RWA at 650 nm, to an absorption‐dominated total energy balance at 670 nm. At the latter wavelength and |θ|=40°, the calculated contrasts satisfy $C_{T}≃C_{A}$ and $C_{R}≃0$, whereas higher‐order transmitted diffraction channels remain propagating in the ${\text{SiO}}_{2}$ substrate so that the complete scattering problem remains multiport. The corresponding outgoing‐field and normalized local‐loss distributions provide spatial corroboration of this channel‐resolved energy balance. The principal asymmetric spectral evolution is examined experimentally using the ${\text{SiO}}_{2}$–Ag platform and is further evaluated in a directionally Al‐coated PMMA–${\text{TiO}}_{2}$ grating on a ${\text{SiO}}_{2}$ substrate. These results establish a channel‐based physical framework and a transferable design strategy for reciprocal lateral AT in symmetry‐broken metal–dielectric gratings.

## 2 Design and Theoretical Framework

Figure 1 conceptually illustrates the material arrangement, broken lateral mirror symmetry, and reciprocal $+k_{x}$ and $-k_{x}$ excitation configurations. It represents the same directionally Ag‐coated ${\text{SiO}}_{2}$ grating platform investigated experimentally in Figure 2, but it is not intended as a to‐scale reconstruction of the fabricated cross section. The experimental structure consists of a one‐dimensional ${\text{SiO}}_{2}$ grating on a ${\text{SiO}}_{2}$ substrate, with directionally deposited Ag forming a continuous region over the ridge top, the top‐to‐side corner, and predominantly one ridge sidewall. The morphology‐informed RCWA model has a grating period of P=400nm, an effective ${\text{SiO}}_{2}$ ridge‐core width of W≃166nm, and an effective ridge height of H≃344nm. The maximum lateral extent of the continuous metal‐covered profile is $W+a_{c}≃199\;nm$. The complete cross‐sectional parameterization is provided in Supporting Information S1: Section S3.

**FIGURE 1:**
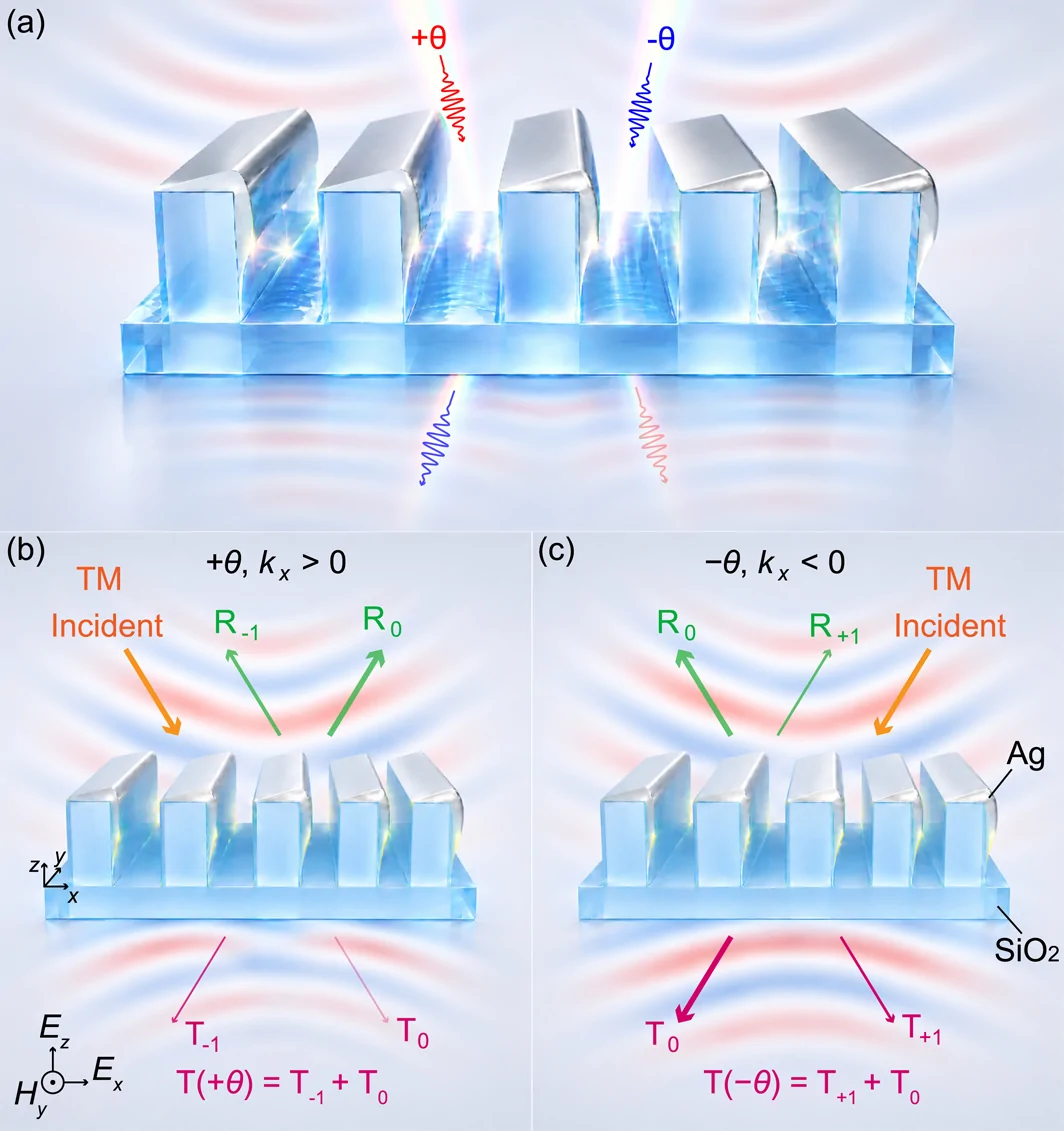
Geometry and reciprocal lateral‐excitation configurations of the directionally metallized ${\text{SiO}}_{2}$ nanograting. (a) Conceptual overview of the fixed directionally Ag‐coated grating. The red and blue incident‐wave trajectories distinguish the +θ and −θ configurations, respectively; the colors serve only as visual identifiers of the two configurations. (b) Separate diffraction‐channel schematic under TM‐polarized +θ incidence with $k_{x}>0$. (c) Separate diffraction‐channel schematic under TM‐polarized −θ incidence with $k_{x}<0$. In panels (b) and (c), the orange, green, and magenta arrows denote the incident, reflected, and transmitted waves, respectively, and $R_{m}$ and $T_{m}$ identify the corresponding reflected and transmitted diffraction orders. The grating geometry and the orientation of the one‐sided Ag coating remain unchanged between the two configurations, and both beams illuminate the same fixed grating from the same upper air half‐space. For each configuration, the total transmittance is obtained by summing the efficiencies of all propagating transmitted diffraction orders. The lateral transmission contrast is defined as $C_{T}=T\left(-\theta \right)-T\left(+\theta \right)$. For each configuration, the total transmittance is obtained by summing the efficiencies of all propagating transmitted diffraction orders. For the configurations illustrated in panels (b) and (c), this gives $T\left(+\theta \right)=T_{-1}+T_{0}$ and $T\left(-\theta \right)=T_{+1}+T_{0}$, respectively. OpenAI ChatGPT was used in July 2026 solely for visual refinement of this nondata‐bearing conceptual illustration. All scientific details were verified by the authors.

**FIGURE 2:**
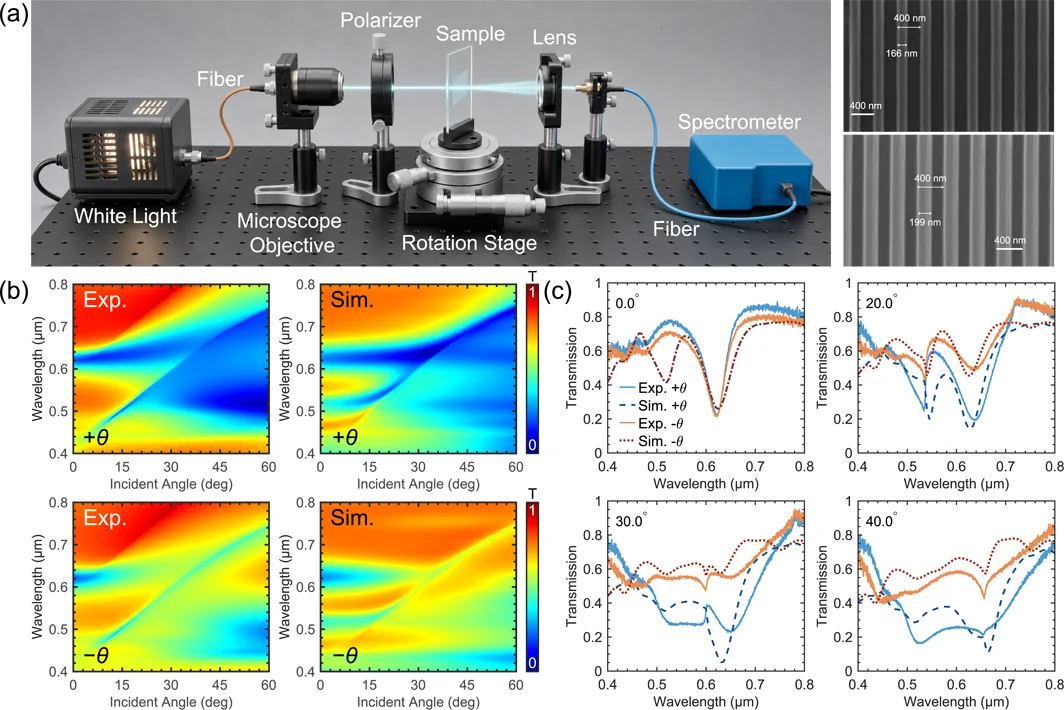
Experimental characterization and comparison with the morphology‐informed RCWA model. (a) Schematic of the angle‐resolved transmission measurement setup. The upper and lower top‐view SEM images show the ${\text{SiO}}_{2}$ grating before and after directional Ag deposition, respectively; both scale bars are 400 nm. (b) Experimentally measured (Exp.) and RCWA‐calculated (Sim.) maps of the total transmission T as functions of wavelength and the absolute incidence angle. The upper and lower rows correspond to the +θ and −θ configurations, respectively, and all maps use the same color scale, 0≤T≤1. (c) Experimental and calculated transmission spectra at incidence angles of 0°, 20°, 30°, and 40°. The blue solid and dashed curves denote the experimental and calculated results for the +θ configuration, respectively, whereas the orange solid and red dotted curves denote the corresponding results for the −θ configuration. All calculations use the morphology‐informed model with a grating period of P=400nm and an effective ${\text{SiO}}_{2}$ ridge‐core width of W≃166nm; W denotes the ${\text{SiO}}_{2}$ core width rather than the maximum lateral extent of the complete Ag‐coated profile. OpenAI ChatGPT was used in July 2026 solely for the visual refinement of the measurement‐setup schematic portion of panel (a). All scientific details were verified by the authors.

The continuous one‐sided Ag contour is physically informed by the oblique‐deposition morphology reported in ref. [29], where top‐view and cross‐sectional SEM observations show that directional metal deposition can produce accumulation over the ridge top and corner together with a finite coverage of the sidewall facing the deposition source. This reference is used only to support the physical plausibility of the adopted morphology class; its geometrical dimensions and deposition conditions are not transferred to the present model. The parameters of the present ${\text{SiO}}_{2}$–Ag model were independently constrained using the available structural information and measured angular–spectral transmission response and are therefore not interpreted as a unique microscopic reconstruction of the fabricated sample. The ${\text{SiO}}_{2}$ grating and substrate are modeled as lossless dielectric media with $n_{SiO_{2}}=1.46$ over the investigated wavelength range. The wavelength‐dependent optical response of Ag is described using the Lorentz–Drude model [30]. To account phenomenologically for unresolved departures of the directionally deposited Ag from an ideal dense film, constrained Drude‐damping and local optical‐dilution corrections are applied only within the deposited‐Ag region. The complete material formulation, parameter values, and interpretation limits are provided in the Supporting Information S1. A Cartesian coordinate system is defined such that the grating periodicity is along the x direction, the invariant direction is along y, and the surface normal is along z. The incidence angle θ is measured in the x–z plane with respect to the surface normal. All results presented in this work correspond to TM‐polarized incidence for which the electric field lies in the plane of incidence, $E^{\text{TM}}=\left(E_{x},0,E_{z}\right)$, and the magnetic field is oriented along the grating grooves, $H^{\text{TM}}=\left(0,H_{y},0\right)$. The +θ and −θ configurations have the same TM polarization and differ only in the sign of the in‐plane incident wavevector $k_{x}$. Throughout this work, lateral AT refers to the difference between the total transmitted powers under $+k_{x}$ and $-k_{x}$ excitation. Both beams illuminate the same fixed grating from the same upper air half‐space and propagate toward the ${\text{SiO}}_{2}$ substrate, with opposite in‐plane wavevector components and the same normal wavevector component. The two configurations therefore probe the broken lateral mirror symmetry of the directionally metallized unit cell under fixed TM polarization. They should not be confused with top–bottom, or vertical, AT obtained by illuminating the structure from opposite half‐spaces, which is not investigated here.

The directionally deposited Ag profile breaks the lateral mirror symmetry of the unit cell, $\varepsilon_{r}\left(x,z\right)\neq \varepsilon_{r}\left(-x,z\right)$, whereas the constituent materials remain linear, passive, and nonmagnetic. The structure therefore remains reciprocal. In a power‐normalized basis with reciprocal diffraction channels consistently paired, Lorentz reciprocity requires

(1) $$S\left(k_{x}\right)=S^{T}\left(-k_{x}\right).$$

This relation connects the scattering amplitudes of individual reciprocal channel pairs. Broken lateral mirror symmetry allows the $+k_{x}$ and $-k_{x}$ configurations to couple differently to the structure, but symmetry breaking alone does not guarantee unequal total transmission. If only one reflected and one transmitted propagating channel are available for the fixed polarization, the system is effectively two‐port and reciprocity enforces equal transmission between reciprocal channels. When higher‐order diffraction channels become propagating, the system becomes multiport, and Equation (1) does not generally impose equality on the total transmitted powers summed over all propagating diffraction orders. For each incidence direction, energy conservation requires T(θ)+R(θ)+A(θ)=1. We therefore define the directional transmission contrast as

(2) $$C_{T}=T\left(-\theta \right)-T\left(+\theta \right)=C_{R}+C_{A},$$

where $C_{R}=R\left(+\theta \right)-R\left(-\theta \right)$ and $C_{A}=A\left(+\theta \right)-A\left(-\theta \right)$ denote the corresponding reflection and absorption contrasts, respectively. A positive $C_{T}$ indicates greater total transmission for the −θ configuration, whereas positive $C_{R}$ and $C_{A}$ indicate greater reflection and absorption, respectively, for the +θ configuration. This decomposition is used strictly as an energy‐balance diagnostic. It identifies whether the direction‐dependent transmission difference is compensated predominantly by reflection, absorption, or both; it does not treat reflection or absorption as an independent origin of lateral AT.

For diffraction order m, the tangential component is $k_{x,m}=k_{0}\;\sin\;\theta +mG$, with G=2π/P. In medium j, the normal component is $q_{m}^{\left(j\right)}=\sqrt{{\left(n_{j}k_{0}\right)}^{2}-k_{x,m}^{2}}$. Although $k_{x,m}$ is conserved across the interfaces, the normal wavevector components $q_{m}^{\left(air\right)}$ and $q_{m}^{\left(sub\right)}$ generally differ because $n_{\text{air}}\neq n_{\text{sub}}$. In each lossless surrounding medium, an order is propagating when $q_{m}^{\left(j\right)}$ is real and evanescent when $q_{m}^{\left(j\right)}$ is imaginary. The corresponding RWA occurs at the medium‐specific cutoff condition $q_{m}^{\left(j\right)}=0$. Consequently, a higher‐order transmitted diffraction channel can remain propagating in the higher‐index ${\text{SiO}}_{2}$ substrate, whereas the corresponding reflected order is evanescent in air. The propagating reflected orders in air and the propagating transmitted orders in the substrate must therefore be evaluated separately, and the total reflectance and transmittance are obtained by summing over their respective open‐order sets. Under the reciprocal $+k_{x}$ and $-k_{x}$ configurations, individual diffraction orders are mapped onto their reciprocal counterparts, whereas the laterally asymmetric structure can redistribute power differently among the open reflected and transmitted channels and the absorptive channel.

The optical response is calculated using rigorous coupled‐wave analysis (RCWA) [31, 32] without homogenizing the periodic ${\text{SiO}}_{2}$–Ag grating into a single effective propagation medium. This treatment avoids invoking an effective‐medium approximation near the diffraction and resonant features of a high‐contrast periodic grating, where homogenized descriptions can become unreliable [33]. The morphology‐informed unit cell is represented by an explicit, piecewise spatially varying, and wavelength‐dependent permittivity distribution $\varepsilon_{r}\left(x,z;\lambda \right)$, which is expanded along the periodic x direction and stratified along z. The permittivity and TM field expansions are

(3) $$\begin{array}{cc} \;\varepsilon_{r}\left(x,z;\lambda \right)\; & =\sum_{p}\varepsilon_{p}\left(z;\lambda \right)e^{\mathrm{ipGx}},\\ \;F_{k_{x}}\left(x,z\right)\; & =\sum_{m}F_{m}\left(z\right)e^{i\left(k_{x}+mG\right)x}. \end{array}$$

here $\varepsilon_{p}\left(z;\lambda \right)$ denotes the pth Fourier coefficient of the spatially varying permittivity, and $F_{m}\left(z\right)$ denotes the corresponding TM field harmonic. The coupled Fourier amplitudes are solved directly for the morphology‐informed asymmetric unit cell. No single effective refractive index is assigned to the periodic grating.

The field expansion in Equation (3) follows from Bloch–Floquet periodicity. Momentum‐resolved Bloch‐mode analysis provides an appropriate framework for describing wave propagation and dispersion in periodic photonic structures [34]. Because the present structure is periodic along x with period P, the TM field satisfies

(4) $$F_{k_{x}}\left(x+P,z\right)=e^{ik_{x}P}F_{k_{x}}\left(x,z\right).$$

Equivalently, $F_{k_{x}}\left(x,z\right)=e^{ik_{x}x}u_{k_{x}}\left(x,z\right)$, where $u_{k_{x}}\left(x+P,z\right)=u_{k_{x}}\left(x,z\right)$. Because the incident medium is air, the conserved Bloch wavevector is $k_{x}=k_{0}\;\sin\;\theta$ modulo G. The +θ and −θ configurations therefore correspond to opposite signs of $k_{x}$, whereas the physical grating and the orientation of the one‐sided Ag coating remain fixed. The harmonics $k_{x}+mG$ define the diffraction channels of the periodic scattering problem, and their propagating or evanescent character is determined separately in air and in the ${\text{SiO}}_{2}$ substrate from the corresponding medium‐dependent normal wavevector components $q_{m}^{\left(j\right)}$.

The angular–spectral response calculated by RCWA is a driven Bloch–Floquet scattering response at real frequencies. Because the structure is vertically open and contains dispersive and absorptive Ag, the observed spectral branches are described as driven Bloch–Floquet scattering features rather than as lossless closed‐system Bloch eigenbands. The lateral transmission contrast is attributed to direction‐dependent coupling and power redistribution among the propagating diffraction channels under reciprocal $+k_{x}$ and $-k_{x}$ excitation, not to a nonreciprocal splitting of the Bloch dispersion. RCWA is used here as a systematically refinable full‐wave solution of the macroscopic frequency‐domain Maxwell equations for a specified morphology‐informed geometry and dispersive material response; it is not a parameter‐free first‐principles calculation. Details of the numerical implementation, refinement assessment, modeling assumptions, and interpretation limits are provided in Supporting Information S1: Section S3.

## 3 Results and Discussion

Figure 2 presents the experimental characterization of the directionally Ag‐coated ${\text{SiO}}_{2}$ nanograting and its comparison with the morphology‐informed RCWA model. The sample corresponds to the same laterally asymmetric ${\text{SiO}}_{2}$–Ag grating platform represented conceptually in Figure 1, whereas Figure 1 is not intended as a to‐scale reconstruction of the fabricated cross section. The angle‐resolved transmission setup in Figure 2a employs a broadband white‐light source, TM‐polarized illumination, a sample mounted on a rotation stage, and fiber‐coupled spectral detection. The +θ and −θ measurements correspond to the two reciprocal lateral excitation configurations defined in Figure 1. The top‐view SEM images support a grating period close to 400 nm and the deposition‐induced lateral modification of the grating morphology.

The morphology‐informed RCWA model used for Figure 2 has a period of P=400nm, an effective ${\text{SiO}}_{2}$ ridge‐core width of W≃166nm, and an effective ridge height of H≃344nm. Directional Ag deposition is represented by one continuous top/corner/one‐sided‐sidewall region with a maximum lateral extent of $W+a_{c}≃199\;nm$. The one‐sided morphology class is physically informed by the authors in ref. [29], whereas the geometrical dimensions of that reference are not used for the present sample. The present parameters were independently constrained using the available structural information and measured angular–spectral transmission response. They therefore define an effective optical cross section that is consistent with the experimental response under the imposed constraints, rather than a unique microscopic reconstruction of the fabricated sample. Complete model definitions and parameters are provided in Supporting Information S1: Section S3. The period P determines the kinematic cutoff of the diffraction orders through the Fourier–Floquet momentum condition, whereas the ridge‐core width W affects the corresponding Fourier coupling strengths, spectral weights, and resonance‐assisted redistribution among the available channels. For P=400nm and |θ|=40°, the air‐side first‐order RWA occurs at $\lambda_{\text{RWA}}^{\left(air\right)}=P\left(1+\sin\;40{}^{\circ}\right)≃657\;nm$. This cutoff corresponds to the m=−1 reflected order for the +θ configuration and the reciprocal m=+1 reflected order for the −θ configuration. Because the lower half‐space is ${\text{SiO}}_{2}$, the propagating transmitted orders and their cutoff conditions are evaluated separately using the substrate refractive index.

The measured and calculated angular–spectral transmission maps in Figure 2b exhibit the same principal evolution. Near normal incidence, the two lateral excitation configurations show nearly overlapping measured spectra, while the calculated +θ and −θ responses coincide. As the incidence angle increases, the long‐wavelength transmission band becomes progressively different for the two configurations, with the +θ response generally exhibiting lower transmission than the corresponding −θ response over the principal asymmetric band. Within the measured wavelength and angular ranges, the largest experimental transmission contrast is $C_{T}≃0.431$ at λ≃648.7 nm and |θ|=59.4°, where T(+θ)≃0.278 and T(−θ)≃0.709. At the nearest simulated grid point, λ=650 nm and |θ|=59.4°, the model gives T(+θ)≃0.236, T(−θ)≃0.691, and $C_{T}≃0.455$.

The fixed‐angle spectra in Figure 2c further show that the morphology‐informed model reproduces the sign of the lateral transmission difference and the overall angular evolution of the principal spectral features at 0°, 20°, 30°, and 40°. Some quantitative deviations remain in the resonance amplitudes, linewidths, and spectral positions, particularly at larger incidence angles. The comparison is therefore interpreted as consistency in the principal spectral evolution and lateral‐transmission‐contrast trend under the imposed constraints, rather than as an exact or unique reconstruction of every experimental feature. Figure 2 thus provides the experimental and numerical basis for the order‐resolved energy and field analysis presented in Figure 3.

**FIGURE 3:**
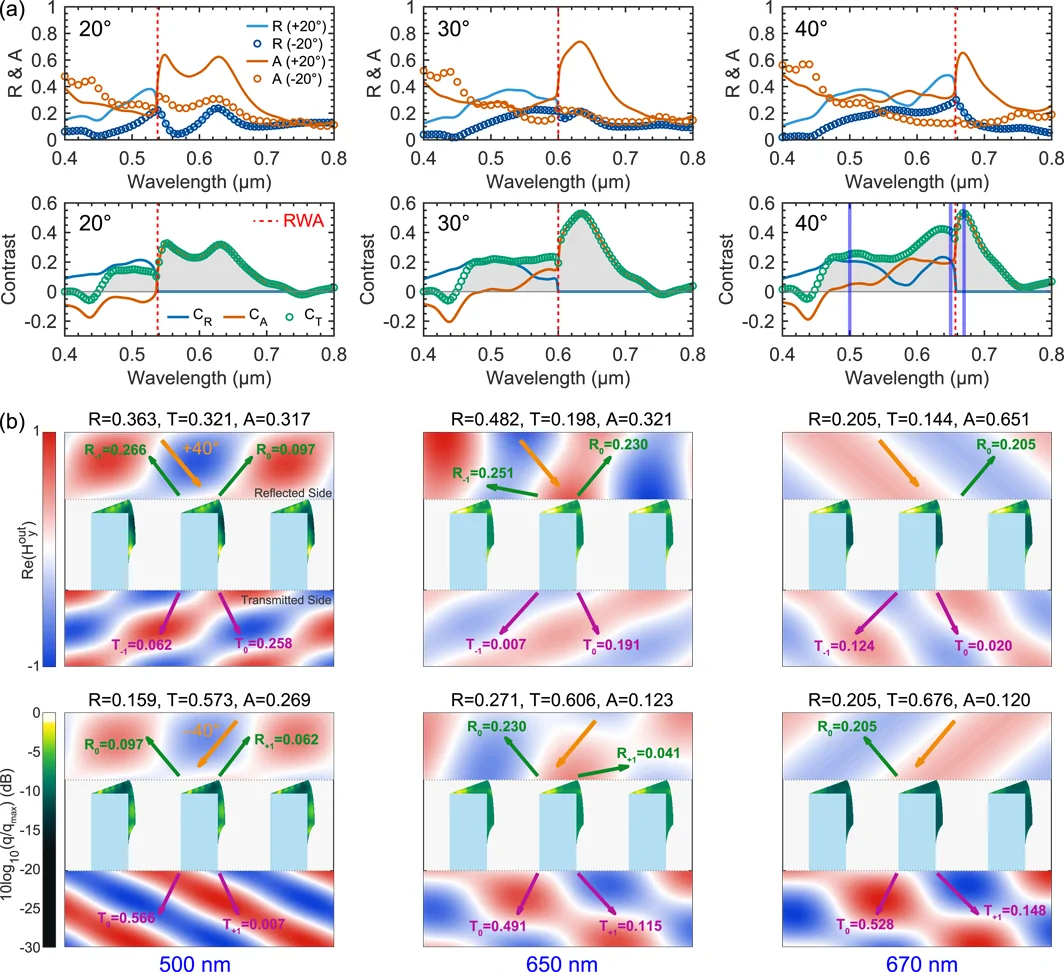
Order‐resolved energy and field analysis of lateral asymmetric transmission. (a) Direction‐dependent reflectance and absorptance at θ=20°, 30°, and 40°, together with the contrast decomposition $C_{T}=C_{R}+C_{A}$. The red dash‐dotted lines labeled “RWA” indicate the air‐side Rayleigh–Wood anomalies associated with the cutoff of the relevant first‐order reflected diffraction channel. The solid vertical markers in the 40° contrast panel identify 500, 650, and 670 nm. (b) Outgoing‐field and local‐loss distributions at θ=±40° for 500, 650, and 670 nm. The upper and lower rows correspond to +40° and −40° incidence, respectively. The red–blue distributions show the normalized $\text{Re}\;\left(H_{y}^{\text{out}}\right)$ in the reflected and transmitted regions. Orange, green, and magenta arrows indicate the incident, reflected, and transmitted wavevector directions, respectively, and the labels give the efficiencies of the corresponding diffraction orders. The colored distributions within Ag show $10\log_{10}\left(q/q_{\max}\right)$, where $q=\left(\pi /\lambda \right)\text{Im}\;\left(\varepsilon_{r}\right)\left(|E_{x}{|}^{2}+|E_{z}{|}^{2}\right)$ is a scaled local‐loss indicator proportional to the time‐averaged Ohmic‐loss density. For each wavelength, the +40° and −40° configurations use the same field and loss normalization.

To determine how the direction‐dependent multiport power redistribution is manifested in the total energy balance, Figure 3 presents an order‐resolved energy and field analysis. Figure 3a compares the total reflectance and absorptance for the reciprocal ±θ configurations and decomposes the transmission contrast according to Equation (2). At 500 nm and |θ|=40°, an additional first‐order reflected diffraction channel is propagating in air. The calculated contrasts are $C_{T}≃0.252$, $C_{R}≃0.204$, and $C_{A}≃0.048$. The transmission contrast is therefore predominantly compensated by the difference in total reflection at this wavelength. Near the air‐side RWA at 650 nm, the reflection and absorption contributions become comparable with $C_{T}≃0.408$, $C_{R}≃0.210$, and $C_{A}≃0.198$. This intermediate regime marks the evolution from a predominantly reflection‐compensated total energy balance toward one with a substantial direction‐dependent absorptive contribution. At 670 nm, on the long‐wavelength side of the air‐side RWA cutoff, only the specular reflected order remains propagating in air. The total reflectances are R(+40°)=R(−40°)≃0.205, and hence $C_{R}≃0$. Higher‐order transmitted diffraction channels nevertheless remain propagating in the higher‐index ${\text{SiO}}_{2}$ substrate. For +40° incidence, the transmitted‐order efficiencies are $T_{-1}=0.124$ and $T_{0}=0.020$, giving T(+40°)=0.144. For −40° incidence, the corresponding efficiencies are $T_{0}=0.528$ and $T_{+1}=0.148$, giving T(−40°)=0.676. According to energy conservation, the corresponding contrasts satisfy $C_{T}≃C_{A}≃0.53$ and $C_{R}≃0$. Thus, nearly identical total reflectances do not imply equal total transmittance. The lateral transmission contrast at 670 nm is absorption‐dominated in the total energy balance, whereas the complete scattering problem remains multiport because of the propagating higher‐order transmission channels in the substrate.

Figure 3b provides the corresponding spatial evidence. The normalized $\text{Re}\;\left(H_{y}^{\text{out}}\right)$ distributions visualize the phase patterns of the propagating reflected and transmitted diffraction orders, whereas the arrows and labeled efficiencies identify their wavevector directions and energy contributions. Because $\text{Re}\;\left(H_{y}^{\text{out}}\right)$ represents the signed phase pattern of the outgoing field, it is not itself used as a measure of transported power. Under the field normalization used in the RCWA calculation, the scaled local‐loss indicator is defined as $q\left(x,z\right)=\left(\pi /\lambda \right)\text{Im}\;\left[\varepsilon_{r}\left(x,z;\omega \right)\right]\left(|E_{x}\left(x,z\right){|}^{2}+|E_{z}\left(x,z\right){|}^{2}\right)$ and is proportional to the time‐averaged Ohmic‐loss density. The loss distributions are plotted as $10\log_{10}\left(q/q_{\max}\right)$; therefore, the omitted global proportionality constant does not affect the normalized maps. For each wavelength, the two reciprocal configurations use the same normalization. At 670 nm, the substantially stronger Ag loss under +40° incidence is consistent with the substantially larger total absorptance obtained for this configuration than for −40° incidence. The loss distributions therefore provide spatial corroboration of the order‐resolved energy balance rather than replacing the quantitative absorption calculation.

Taken together, these results show that higher‐order diffraction provides the multiport channel space required for polarization‐preserving lateral AT, whereas $C_{R}$ and $C_{A}$ diagnose how the resulting direction‐dependent power redistribution is manifested in the total energy balance. Each RWA marks a medium‐specific diffraction‐order cutoff rather than an independent localized or plasmonic resonance. The SPP‐related, localized‐plasmonic, and hybrid metal–dielectric resonant responses of the present structure therefore do not constitute independent fundamental origins of the asymmetry. Instead, they modify the coupling strengths among the open diffraction channels and thereby control the spectral evolution of $C_{T}$, the spatial field localization, and the accompanying direction‐dependent metallic dissipation. The response at 500 nm is consequently described as predominantly reflection‐compensated, the response near the RWA at 650 nm as jointly compensated by reflection and absorption, and the response at 670 nm as absorption‐dominated in the total energy balance. These classifications describe different manifestations of the energy balance within the same multiport mechanism rather than three distinct physical origins of lateral AT.

To examine whether the lateral‐asymmetry design principle is restricted to the ${\text{SiO}}_{2}$–Ag platform, we further investigated a second material system consisting of directionally Al‐coated ${\text{TiO}}_{2}$ ridges embedded in a PMMA layer on a ${\text{SiO}}_{2}$ substrate. The one‐sided Al profile breaks the lateral mirror symmetry of the unit cell, whereas the +θ and −θ configurations correspond to opposite in‐plane wavevectors under the same TM polarization. The calculated spectra were obtained using the morphology‐informed RCWA model described in Supporting Information S1: Section S3. As for the ${\text{SiO}}_{2}$–Ag structure, the reported parameter set should be regarded as an effective cross‐sectional model that is consistent with the measured spectra under the imposed geometrical and material constraints, rather than as a unique microscopic reconstruction of the fabricated sample.

Figure 4a compares the measured and calculated angle‐resolved total‐transmission maps. Both experiment and simulation exhibit a clear separation between the two lateral excitation configurations in the long‐wavelength region, where T(−θ) is consistently higher than T(+θ) over a broad angular range. The fixed‐angle spectra in Figure 4b further show that this transmission contrast generally becomes more pronounced with the increasing incidence angle. For example, at λ=675nm and |θ|=50°, the measured transmissions are T(+θ)≃0.334 and T(−θ)≃0.513, corresponding to $C_{T}≃0.179$. The calculated values are T(+θ)≃0.341 and T(−θ)≃0.522, giving $C_{T}≃0.181$.

**FIGURE 4:**
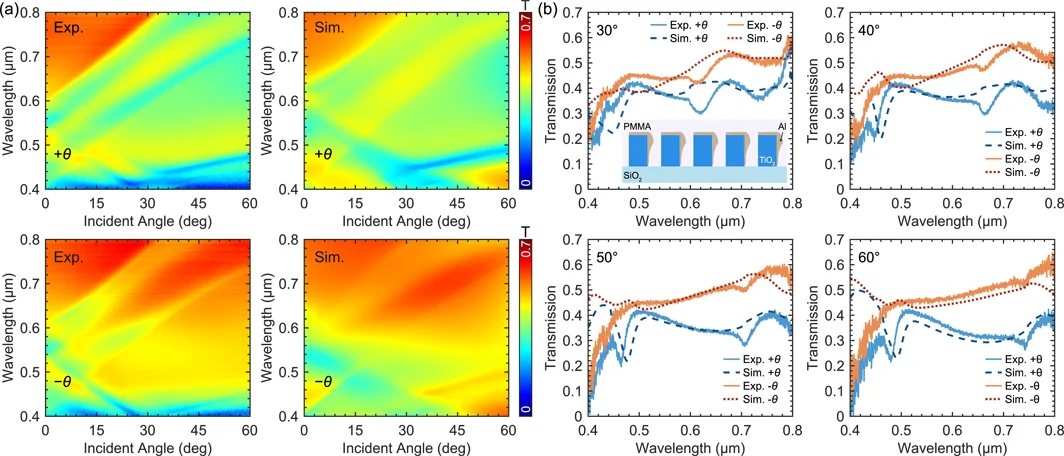
Experimental and numerical examination of lateral asymmetric transmission in a directionally Al‐coated PMMA–${\text{TiO}}_{2}$ grating on a ${\text{SiO}}_{2}$ substrate. (a) Measured (Exp.) and morphology‐informed RCWA‐calculated (Sim.) angle‐resolved total‐transmission maps for the +θ configuration (top row) and the −θ configuration (bottom row). All four maps use the same color scale, 0≤T≤0.7. (b) Experimental and calculated transmission spectra at |θ|=30°, 40°, 50°, and 60°. The solid light‐blue and dashed dark‐blue curves represent the experimental and calculated results for +θ, respectively, whereas the solid orange and dotted dark‐red curves represent the corresponding results for −θ. The inset schematically illustrates the PMMA–${\text{TiO}}_{2}$–Al cross‐section. The complete geometry, material parameters, and numerical settings of the morphology‐informed model are provided in Supporting Information S1: Section S3.

The morphology‐informed calculation therefore reproduces the sign of the transmission contrast, the broad long‐wavelength separation between the two excitation configurations, and its principal angular evolution. Quantitative differences remain in the detailed spectral line shapes and in the magnitude of the contrast, particularly at the largest incidence angles. Nevertheless, the observation of the same lateral transmission‐contrast tendency in a PMMA–${\text{TiO}}_{2}$–Al platform indicates that the design strategy is not restricted to the specific ${\text{SiO}}_{2}$–Ag material combination. Instead, broken lateral mirror symmetry and diffraction‐mediated redistribution among the available optical channels constitute transferable design ingredients, whereas the detailed spectral positions and attainable contrast depend on the particular geometry and material response.

## 4 Conclusions

In summary, we have established a channel‐resolved physical picture of polarization‐preserving reciprocal lateral AT in directionally Ag‐coated ${\text{SiO}}_{2}$ nanogratings. The one‐sided metal morphology breaks the lateral mirror symmetry and allows the reciprocal $+k_{x}$ and $-k_{x}$ excitation configurations to couple differently to the structure, whereas propagating higher‐order diffraction channels provide the required multiport scattering environment. Structure‐dependent plasmonic, cavity‐like, and localized field responses subsequently modify the redistribution of optical power among the open diffraction and absorptive channels, rather than acting as an independent origin of the asymmetry. The order‐resolved energy analysis shows that the transmission contrast evolves from a predominantly reflection‐compensated response at 500 nm to a mixed reflection–absorption regime near the air‐side RWA at 650 nm and an absorption‐dominated total energy balance at 670 nm. At 670 nm and |θ|=40°, $C_{T}≃C_{A}≃0.53$ and $C_{R}≃0$, even though higher‐order transmitted diffraction channels remain propagating in the ${\text{SiO}}_{2}$ substrate. The corresponding field and local‐loss distributions spatially corroborate the direction‐dependent channel redistribution and metallic dissipation.

Angle‐resolved measurements confirm strong lateral transmission contrast in the fabricated ${\text{SiO}}_{2}$–Ag grating. The largest measured contrast within the investigated range is $C_{T}≃0.431$ at λ≃648.7nm and |θ|=59.4°, whereas the morphology‐informed model gives $C_{T}≃0.455$ at the nearest simulated grid point. The model reproduces the sign and principal angular–spectral evolution of the measured contrast, although quantitative differences remain in some resonance positions, linewidths, and amplitudes. A second PMMA–${\text{TiO}}_{2}$–Al platform exhibits the same long‐wavelength lateral‐transmission tendency; at λ=675nm and |θ|=50°, the measured and calculated contrasts are approximately 0.179 and 0.181, respectively. These results indicate that broken lateral mirror symmetry and diffraction‐mediated multiport redistribution are transferable design ingredients rather than features restricted to a single material combination. The resulting response may provide a compact basis for angular spectral filtering, discrimination of the sign of the incident in‐plane wavevector, reciprocal multiport beam routing, and channel‐selective attenuation. When combined with an appropriate absorbing, photosensitive, or active layer, the direction‐dependent absorptive response may also be relevant to angle‐selective sensing and photodetection. These possibilities are prospective, and their practical realization will require further optimization of the operating wavelength, spectral bandwidth, angular tolerance, material loss, and collection or absorption of the selected diffraction channels.

## Author Contributions

**Ye Ming Qing:** conceptualization, validation, investigation, formal analysis, supervision, writing – review and editing, writing – original draft, visualization, funding acquisition, data curation. **Yi Shen:** conceptualization, writing – review and editing, methodology, data curation, investigation. **Shunsuke Murai:** writing – review and editing, funding acquisition, conceptualization. **Katsuhisa Tanaka:** writing – review and editing. **Koichi Okamoto:** writing – review and editing, funding acquisition, conceptualization.

## Conflicts of Interest

The authors declare no conflicts of interest.

## Supporting information


Supporting Information S1

## Data Availability

The data that support the findings of this study are available from the corresponding author upon reasonable request.
