# Supplementary material for: Reciprocal Asymmetric Transmission in Directionally Metallized Silica Nanogratings With Broken Lateral Mirror Symmetry
Source: Nanophotonics. 2026 Aug 8;15(15):e70244. doi: 10.1002/nap2.70244 (PMC13642184; doi:10.1002/nap2.70244)
Supplement: Supplementary file 1 [file NAP2-15-e70244-s001.pdf]

Supplementary Materials

Reciprocal Asymmetric Transmission in Directionally Metallized Silica Nanogratings with Broken Lateral Mirror Symmetry

Ye Ming Qing[1,2,4,*], Yi Shen[3,4], Shunsuke Murai[1,*], Katsuhisa Tanaka[3] and Koichi Okamoto[1,*]

[1] *Department of Physics and Electronics, Osaka Metropolitan University, Osaka 599-8531, Japan*
[2] *College of Electronic and Optical Engineering & College of Flexible Electronics (Future Technology), Nanjing University of Posts and Telecommunications, Nanjing 210023, China*
[3] *Department of Material Chemistry, Kyoto University, Kyoto 615-8510, Japan*
[4] *These authors contributed equally to this work.*
E-mail: ymqing@njupt.edu.cn; murai@omu.ac.jp; OkamoT@omu.ac.jp

---

S1. Sample fabrication

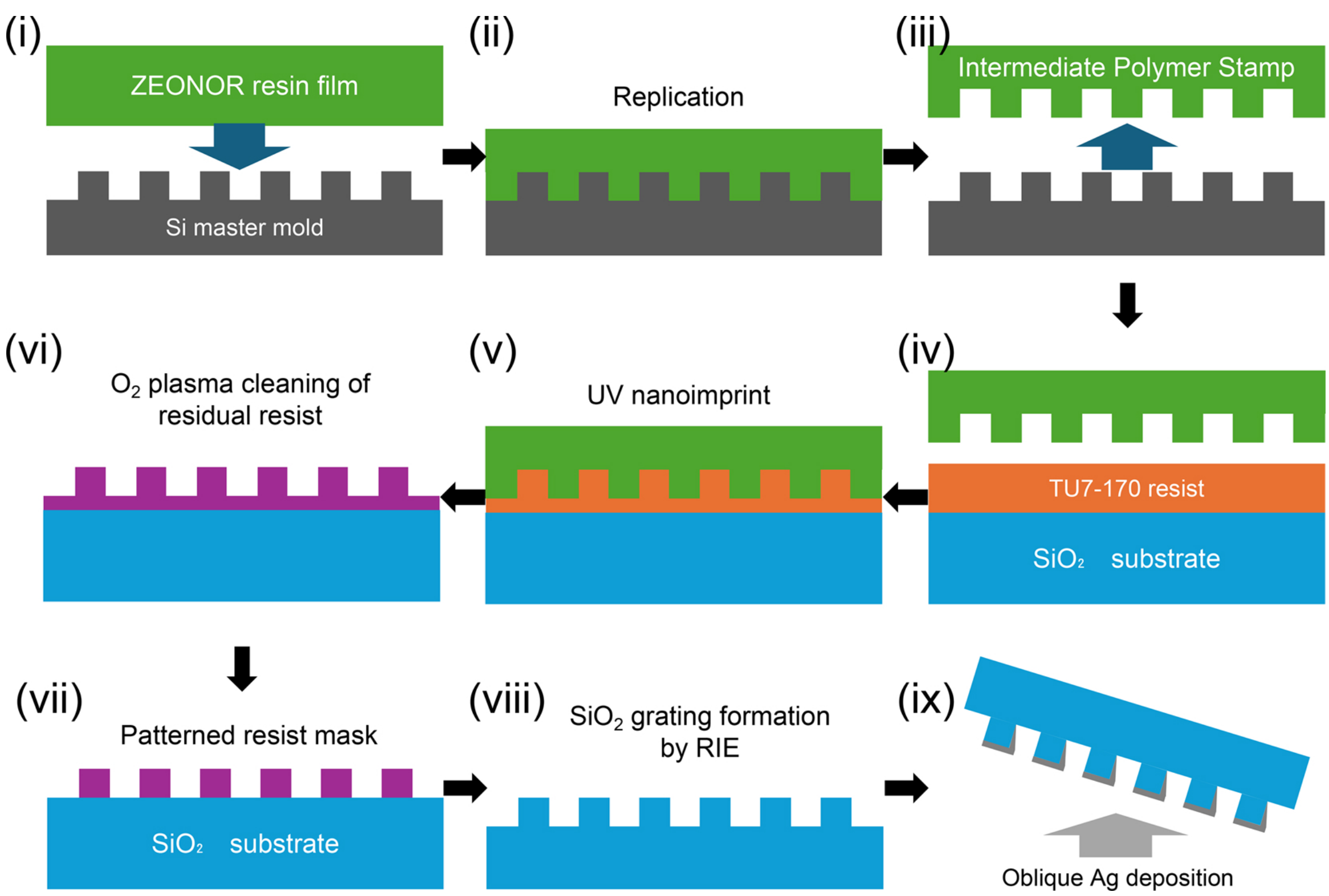

Figure S1. Schematic fabrication process of the laterally asymmetric SiO_2–Ag nanograting. (i)–(iii) Replication of an intermediate polymer stamp from a Si master mold. (iv)–(v) Transfer of the one-dimensional grating pattern into a UV-curable resist layer on a SiO_2 substrate. (vi)–(viii) Removal of the residual resist and transfer of the pattern into the SiO_2 substrate. (ix) Directional Ag deposition, for which geometrical shadowing produces an asymmetric metal profile comprising an upper coating and preferential coverage of one ridge side.

The laterally asymmetric SiO_2–Ag nanograting was fabricated by combining nanoimprint lithography, pattern transfer into SiO_2, and subsequent directional Ag deposition, as illustrated in Fig. S1. A silicon master mold was first prepared by electron-beam lithography (F7000S-KYT01, Advantest, Japan) followed by deep inductively coupled plasma etching (RIE-800iPB-KU, Samco, Japan). An intermediate polymer stamp was then replicated from the master using a ZEONOR resin film.

A thin layer of TU7-170 nanoimprint resist (Obducat, Sweden) was spin-coated onto the SiO_2 substrate and soft-baked. The intermediate polymer stamp was used to transfer the one-dimensional grating pattern into the resist using a UV nanoimprint system (Eitre3, Obducat, Sweden). After imprinting, the residual resist at the groove bottoms was removed by O_2 plasma treatment (RIE-10NR-KF, Samco, Japan). The pattern was subsequently transferred into the SiO_2 substrate

by reactive-ion etching (NLD-570, Ulvac, Japan), and the remaining resist mask was removed. The fabricated grating has a nominal period close to 400 nm.

Ag was then deposited directionally onto the SiO_2 grating using an electron-beam evaporation system (UEP-2000 OT-H/C, Ulvac, Japan). Geometrical shadowing during deposition preferentially coated the ridge top and one lateral side, thereby breaking the mirror symmetry of the unit cell along the grating-period direction. The resulting deposited-metal morphology is non-rectangular and cannot be represented reliably by a uniform top film or an ideal rectangular sidewall layer.

The structural characterization is used to constrain the general morphology of the electromagnetic model used in this work, including the grating period, ridge dimensions, and one-sided shoulder-like metal profile. Because the available images do not uniquely determine all sub-resolution details of the deposited Ag, the fitted geometrical parameters are treated as effective, physically constrained parameters rather than as a unique microscopic reconstruction of the sample. The morphology-informed model used in this work is described in Section S3.

The PMMA–TiO_2–Al sample examined in Fig. 4 was fabricated using an analogous grating-fabrication and directional-metal-deposition sequence. A one-dimensional TiO_2 grating on a SiO_2 substrate was prepared first, after which Al was directionally deposited to produce preferential coverage of the ridge top, the top-to-side corner, and one ridge sidewall. As an additional final step, PMMA was spin-coated over the directionally metallized grating, forming the embedding and cover layer surrounding the TiO_2 ridges and the one-sided Al region. The two experimental platforms therefore share the same directional-metallization principle, while differing in the dielectric-ridge material, deposited metal, and PMMA overlayer. The morphology-informed parameterization of the PMMA–TiO_2–Al structure, including the PMMA-layer thickness, is provided in Section S3.

S2. Angle-resolved transmission measurement

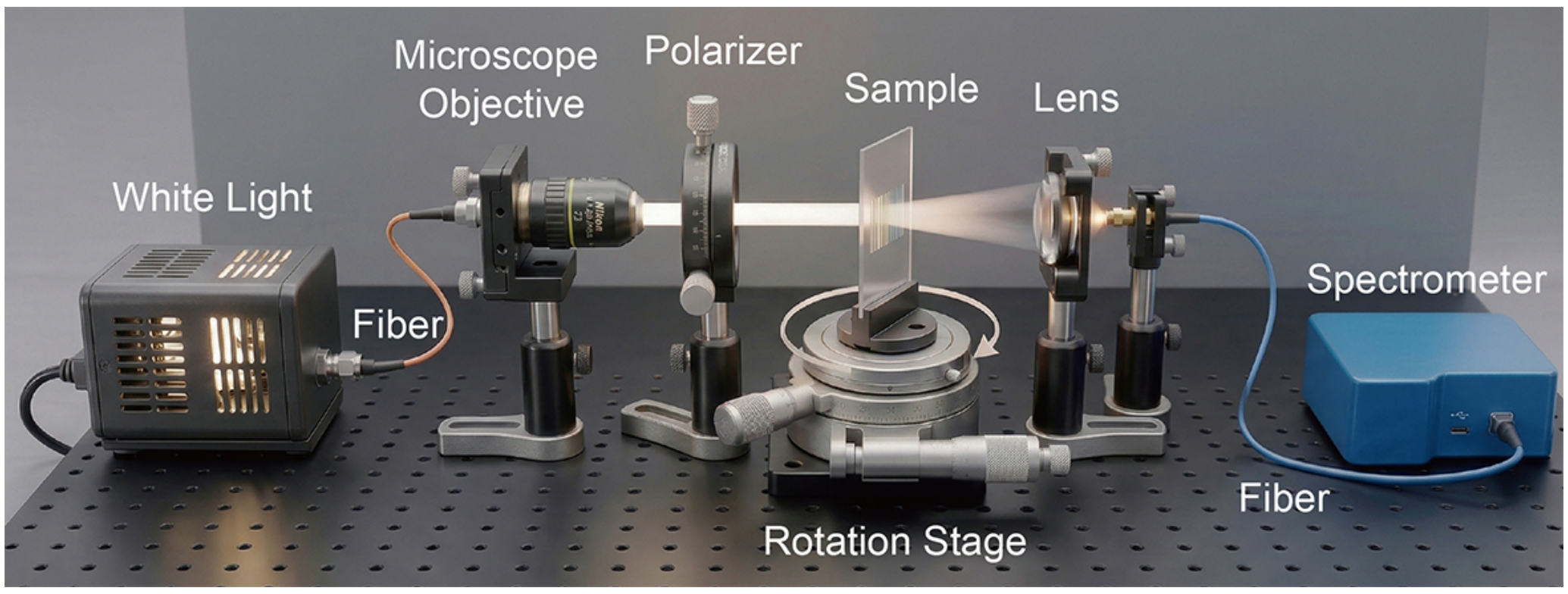

Figure S2. Schematic of the angle-resolved transmission measurement setup.

The angle-resolved transmission spectra were measured using the optical arrangement shown in Fig. S2. A stabilized tungsten–halogen lamp (Thorlabs SLS201L) was used as the broadband light source. The light was coupled into a multimode fiber (Thorlabs M20L01), collimated by a 5× microscope objective (Nikon MUE42050), and linearly polarized using a polarizer (Thorlabs WP25M-VIS). The collimated beam was then directed onto the sample mounted on a rotation stage. The transmitted light was collected by a lens (Thorlabs LB1901), coupled into a multimode fiber (Ocean Optics P400-2-UV-VIS), and recorded using a spectrometer (Ocean Optics FLAME-S).

All spectra discussed in the manuscript were measured under TM-polarized illumination, consistent with the corresponding numerical calculations. For each material platform, illumination was maintained from the same air half-space. Experimentally, the $+\theta$ and $-\theta$ data sets were obtained using the original sample orientation and after azimuthally rotating the sample by 180° about its surface normal, respectively. After mapping both measurements onto a common structural coordinate system referenced to the one-sided metal profile, $+\theta$ denotes $k_x > 0$, whereas $-\theta$ denotes $k_x < 0$. The 180° in-plane rotation reverses the direction of the incident in-plane wavevector relative to the laterally asymmetric unit cell without interchanging the air and substrate half-spaces. The comparison therefore probes two reciprocal lateral excitation configurations rather than illumination from opposite vertical half-spaces.

The spectra were recorded as functions of wavelength and incidence angle. Background signals were removed, and the measured intensities were normalized to reference transmission spectra acquired under the corresponding optical configuration. The same polarization convention and angular definition are used throughout the experiment, numerical calculations, and main-text discussion.

S3. Morphology-informed RCWA model and numerical methodology

The spectra presented in Figs. 2 and 4 were calculated using morphology-informed rigorous coupled-wave analysis (RCWA) models. Both experimental systems were described within the same general geometrical framework: a one-dimensional dielectric grating with a continuous, one-sided deposited-metal region covering the ridge top and one sidewall. The two systems differ in their dielectric and metallic materials, fitted dimensions, and optical environments, but not in the underlying geometrical parameterization.

The geometrical and deposited-metal parameters were determined through constrained fitting to the corresponding angle-resolved transmission spectra. Accordingly, the parameter sets reported below should be regarded as effective morphology-informed descriptions that are consistent with the experimental spectra under the imposed constraints. They are not claimed to represent unique reconstructions of the actual sample morphologies.

For readability, dimensional parameters are reported in integer nanometres whenever practical. The corresponding unrounded values retained in the numerical data files were used in all RCWA calculations.

3.1 Geometry and core parameter sets

Figure S3 places two representative unit-cell cross sections side by side to illustrate the common geometrical parameterization. The left cell represents the SiO_2–Ag model used for Fig. 2, whereas the right cell represents the PMMA–TiO_2–Al model used for Fig. 4. Each structure is modeled independently as a one-dimensional periodic system with period P along the x direction and translational invariance along the grating-groove direction. The coordinate $z = 0$ is defined at the substrate surface, which also corresponds to the base of the dielectric ridge.

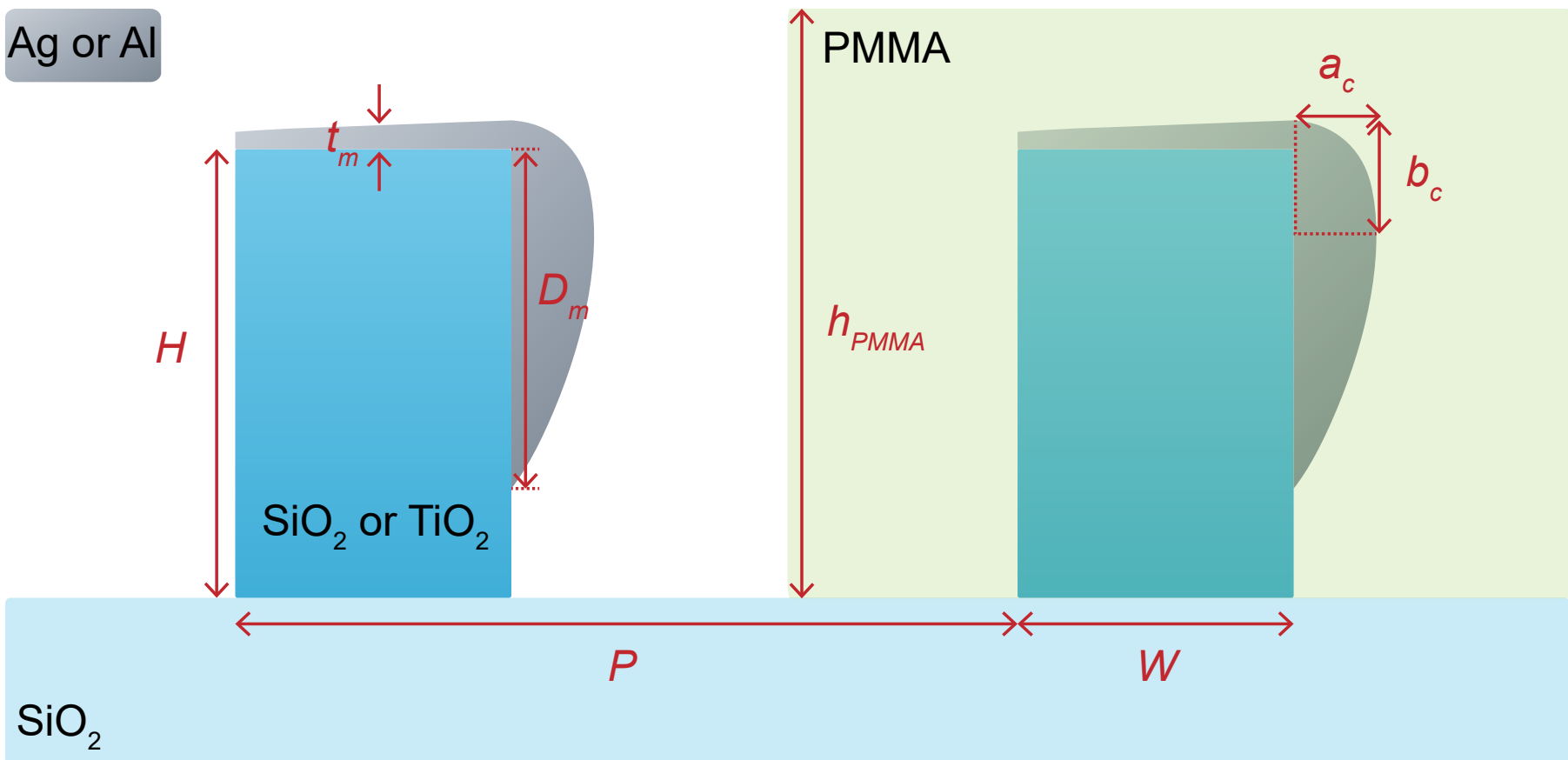

Figure S3. Generic cross-sectional representations of the morphology-informed RCWA models shown side by side for comparison: the SiO_2–Ag model used for Fig. 2 on the left and the PMMA–TiO_2–Al model used for Fig. 4 on the right. The dielectric ridge is described by the period P, ridge width W, and ridge height H. The continuous one-sided deposited-metal region is characterized by the area-equivalent top-metal thickness t_m, side-metal depth D_m, lateral corner extension a_c, and vertical corner-transition span b_c. For the PMMA–TiO_2–Al system, h_{PMMA} denotes the thickness of the PMMA embedding and cover layer measured from the substrate surface to the PMMA–air interface. The PMMA layer is absent from the SiO_2–Ag model. The schematics are not drawn to scale.

The dielectric ridge is described by the grating period P, ridge width W, and ridge height H. The deposited-metal contour is treated as one continuous region covering the ridge top and one sidewall, rather than as separate top and sidewall components. This representation is consistent with the continuous asymmetric morphology expected from directional metal deposition.

Because the detailed top-metal profile may contain a weak thickness variation that cannot be uniquely resolved from the far-field spectra, the reader-facing top-metal thickness is represented by the area-equivalent value

$$t_m = \frac{1}{W} \int_{x_{\text{L}}}^{x_{\text{R}}} t_{\text{top}}(x)\, \mathrm{d}x, \tag{S1}$$

where x_{L} and x_{R} denote the left and right boundaries of the dielectric-ridge top. This definition provides a common and compact description of the top-metal region in both experimental systems, while the corresponding unrounded continuous contours are retained in the numerical calculations.

The side-metal depth is defined as

$$D_m = H - z_{\text{m,b}}, \tag{S2}$$

where $z_{\mathrm{m,b}}$ is the lowest vertical position reached by the continuous side-metal contour. Thus, D_m is measured downward from the dielectric-ridge top to the lower termination of the side coating.

The parameters a_c and b_c describe the lateral and vertical scales of the smooth top-to-side corner transition, respectively. The quantity a_c is also the maximum outward extension of the deposited metal beyond the dielectric-ridge sidewall. Consequently, the maximum lateral extent of the metal-covered ridge is geometrically determined by $W + a_c$ and is not treated as an additional independent parameter.

For the PMMA–TiO_2–Al structure, the TiO_2 ridge and the one-sided Al contour are embedded within a continuous PMMA layer. The parameter h_{PMMA} is measured from the substrate surface to the upper PMMA–air interface. No PMMA layer is included in the SiO_2–Ag model.

The core geometry parameters used for the two experimental systems are summarized in Table S1. The period was fixed according to the grating design, whereas the remaining dimensions represent effective morphology-informed values obtained under the imposed geometrical and spectral constraints. Dimensional parameters are rounded to the nearest nanometre whenever practical; the corresponding unrounded values were used in all RCWA calculations.

Table S1. Core geometry parameters of the morphology-informed RCWA models used for Figs. 2 and 4. Dimensional values are rounded to the nearest nanometre whenever practical.

Quantity	Symbol	SiO_2–Ag	PMMA–TiO_2–Al
Grating period	P	400 nm	400 nm
Dielectric-ridge material	–	SiO_2	TiO_2
Dielectric-ridge height	H	344 nm	120 nm
Dielectric-ridge width	W	166 nm	230 nm
PMMA-layer thickness	h_{PMMA}	–	260 nm
Area-equivalent top-metal thickness	t_m	32 nm	12 nm
Side-metal depth	D_m	344 nm	107 nm
Lowest side-metal position	$z_{\mathrm{m,b}}$	0 nm	13 nm
Maximum lateral corner extension	a_c	33 nm	76 nm
Vertical corner-transition span	b_c	198 nm	42 nm

Although the same geometrical parameterization is used for the two systems, the fitted values are not expected to be identical. The SiO_2–Ag and PMMA–TiO_2–Al samples contain different dielectric ridges, deposited metals, and surrounding dielectric environments, and they were obtained through independent fabrication processes. In particular, the PMMA layer modifies both the optical environment and the effective geometrical constraints of the TiO_2–Al structure.

The differences between the parameter sets therefore should not be interpreted as evidence for fundamentally different deposition mechanisms. Instead, t_m, D_m, a_c, and b_c provide a common set of effective geometrical descriptors for the two independently fitted deposited-metal morphologies. Figure S3 emphasizes this common framework and is not intended to reproduce the relative dimensions to scale.

For the SiO_2–Ag structure, the continuous side-metal contour reaches the substrate surface, giving $z_{\mathrm{m,b}} = 0$ and $D_m \simeq H$. For the PMMA–TiO_2–Al structure, the side-metal contour terminates approximately 13 nm above the substrate surface. The TiO_2 ridge and the Al contour remain embedded within the PMMA layer because $h_{\mathrm{PMMA}} > H$.

3.2 Optical material model

The dielectric regions were represented by real, wavelength-independent refractive indices over the investigated spectral range: $n_{\mathrm{air}} = 1.00$, $n_{\mathrm{SiO_2}} = 1.46$, $n_{\mathrm{PMMA}} = 1.49$, and $n_{\mathrm{TiO_2}} = 2.40$. Thus, the SiO_2, PMMA, and TiO_2 regions were treated as lossless and nondispersive within the present RCWA models, whereas wavelength dispersion and material absorption were retained explicitly in the deposited-metal response. The reported dielectric refractive indices should be understood as effective optical parameters used in the constrained spectral models rather than as independent spectroscopic determinations of the constituent layers.

The optical responses assigned to the deposited Ag and Al regions were described using the wavelength-dependent Lorentz–Drude model reported by Rakić et al. [S1]. Before application of the local optical-dilution correction, the Lorentz–Drude response assigned to the deposited-metal region was calculated as

$$\varepsilon_{\mathrm{m}}^{\mathrm{LD}}(\omega) = 1 - \frac{f_0\omega_{\mathrm{p}}^2}{\omega\left(\omega + \mathrm{i}M_\Gamma\Gamma_0\right)} + \sum_{j=1}^{N_{\mathrm{L}}} \frac{f_j\omega_{\mathrm{p}}^2}{\omega_j^2 - \omega^2 - \mathrm{i}\Gamma_j\omega}, \tag{S3}$$

where ω_{p} is the plasma frequency, f_j, ω_j, and Γ_j are the oscillator strength, resonance frequency, and damping rate of the jth contribution, respectively, and M_Γ is a constrained multiplier applied only to the Drude damping term. The standard

Rakić oscillator parameters used in the calculations are listed in Table S2.

Table S2. Lorentz–Drude parameters used for Ag and Al. The plasma energies, oscillator energies, and damping energies are expressed in electronvolts. The $j = 0$ rows denote the Drude contributions.

Metal	j	$\hbar\omega_p$ (eV)	f_j	$\hbar\Gamma_j$ (eV)	$\hbar\omega_j$ (eV)
Ag	0	9.01	0.845	0.048	–
Ag	1	9.01	0.065	3.886	0.816
Ag	2	9.01	0.124	0.452	4.481
Ag	3	9.01	0.011	0.065	8.185
Ag	4	9.01	0.840	0.916	9.083
Ag	5	9.01	5.646	2.419	20.29
Al	0	14.98	0.523	0.047	–
Al	1	14.98	0.227	0.333	0.162
Al	2	14.98	0.050	0.312	1.544
Al	3	14.98	0.166	1.351	1.808
Al	4	14.98	0.030	3.382	3.473

To phenomenologically account for unresolved deposited-film effects, a local Bruggeman-type correction was applied only within the deposited-metal region [S2]:

$$(1 - f_d)\frac{\varepsilon_m^{LD} - \varepsilon_{dep}}{\varepsilon_m^{LD} + 2\varepsilon_{dep}} + f_d\frac{\varepsilon_b - \varepsilon_{dep}}{\varepsilon_b + 2\varepsilon_{dep}} = 0. \tag{S4}$$

Here, ε_{dep} is the effective dielectric function assigned locally to the deposited-metal region, f_d is an effective optical-dilution parameter, and $\varepsilon_b = 1$ is a low-index reference constituent used in the phenomenological parameterization. The passive solution of Eq. (S4) was selected.

The parameter f_d is not interpreted as a directly measured air-void fraction, oxide fraction, or unique chemical composition. Together with M_Γ, it represents unresolved deposited-film effects, including changes in effective metallic packing, grain-boundary scattering, surface roughness, and other sub-resolution non-metallic contributions. No explicit Al_2O_3 shell is included in the PMMA–TiO_2–Al model.

The optical parameters used in the two models are summarized in Table S3.

Table S3. Optical-material parameters used in the two morphology-informed RCWA models.

Quantity	SiO_2–Ag	PMMA–TiO_2–Al
Air refractive index	1.00	1.00
SiO_2 refractive index	1.46	1.46
PMMA refractive index	–	1.49
TiO_2 refractive index	–	2.40
Deposited metal	Ag	Al
Metal-dispersion model	Rakić Lorentz–Drude	Rakić Lorentz–Drude
Drude damping multiplier, M_Γ	1.15	3.20
Effective optical-dilution parameter, f_d	0.22	0.46
Reference constituent, ε_b	1	1

The local correction in Eq. (S4) modifies only the optical response assigned to the explicitly resolved deposited-metal region. It is not used as an effective-index approximation for the entire grating unit cell.

3.3 RCWA implementation and numerical settings

The structures were treated as one-dimensional periodic systems along x and invariant along the grating-groove direction. Bloch–Floquet periodicity was imposed over one unit cell. The experimentally measured configurations were modeled using TM-polarized illumination from the same upper air half-space with opposite in-plane wavevectors, $+k_x$ and $-k_x$. The physical sample was not inverted between the two excitation configurations.

The continuous deposited-metal contours were discretized into vertically stacked lamellar slices. The tangential electromagnetic-field components at adjacent lamellar interfaces were connected using a numerically stable scattering-matrix formulation. The production calculations used a Fourier truncation parameter of $N = 31$, corresponding to the

retained harmonics from $m = -31$ to $m = +31$, or 63 spatial harmonics in total. The primary vertical discretizations contained 40 contour-control slices for the SiO_2–Ag model and 36 slices for the PMMA–TiO_2–Al model. Representative spectra were additionally recalculated using $N = 51$, corresponding to 103 retained spatial harmonics, together with an increased vertical discretization of 60 slices. Modest quantitative variations were observed in some spectral amplitudes and transmission-contrast values, while the principal spectral features and overall angular trends were preserved.

For each wavelength and incidence angle, the reflected and transmitted diffraction orders were evaluated separately in the upper and lower half-spaces because the two media have different refractive indices and therefore different sets of propagating diffraction channels. The total reflectance and transmittance were calculated by summing the efficiencies of all propagating reflected and transmitted orders, respectively:

$$R = \sum_{m \in O_R} R_m, \qquad T = \sum_{m \in O_T} T_m, \qquad A = 1 - R - T, \tag{S5}$$

where O_R and O_T denote the open reflected- and transmitted-order sets. Evanescent Fourier harmonics were retained in the electromagnetic boundary matching even though they do not directly contribute to the far-field power sums.

3.4 Model assumptions, parameter fitting, and interpretation

The electromagnetic model assumes an infinitely periodic array along x and translational invariance along the grating grooves. The illumination is represented by a monochromatic TM-polarized plane wave, and all constituent materials are described by linear, local, isotropic, and nonmagnetic macroscopic permittivities. The RCWA calculation therefore does not resolve the finite lateral extent of the fabricated array, beam divergence, individual metal grains, microscopic surface roughness, or other sub-resolution morphological variations.

These assumptions can affect the precise resonance wavelengths, linewidths, and absolute spectral amplitudes and can contribute to quantitative differences between calculation and experiment. They do not correspond to weak-coupling or paraxial approximations within the specified periodic model. Numerical errors associated with the Fourier truncation and vertical lamellar discretization are systematically reducible by increasing the number of retained harmonics and slices, as assessed by the refinement comparison described above.

The constrained-fit parameters were adjusted simultaneously within physically restricted ranges to obtain consistency with the measured angle-resolved transmission spectra. The period and material identities were fixed, whereas the ridge dimensions, deposited-metal contour, and deposited-metal optical corrections were allowed to vary within the imposed bounds.

Because the measured far-field spectra do not uniquely determine every sub-resolution geometrical and material feature, correlations can occur among the ridge geometry, metal contour, damping correction, and effective optical-dilution parameter. The parameter sets in Tables S1 and S3 therefore represent compact morphology-informed models that are more consistent with the experimental spectra under the specified constraints. They should not be interpreted as unique structural reconstructions or direct nanoscale chemical measurements.

The common parameterization in Fig. S3 facilitates comparison between the SiO_2–Ag and PMMA–TiO_2–Al systems but does not imply identical deposited-metal dimensions in the two independently fabricated samples. The central conclusions are based on features that do not depend on a unique fitted parameter set: the lateral transmission contrast is observed experimentally; the opening of the propagating diffraction channels follows from the Fourier–Floquet momentum condition and the refractive indices of the exterior media; and the direction-dependent energy redistribution is supported by the order-resolved diffraction efficiencies, the total $R/T/A$ balance, and the corresponding field and q distributions. The model is therefore used to establish a physically constrained and experimentally consistent interpretation rather than a unique microscopic reconstruction.

References

[S1] A. D. Rakić, A. B. Djurišić, J. M. Elazar, and M. L. Majewski, "Optical properties of metallic films for vertical-cavity optoelectronic devices," *Applied Optics* **37**, 5271–5283 (1998).

[S2] D. A. G. Bruggeman, "Berechnung verschiedener physikalischer Konstanten von heterogenen Substanzen. I. Dielektrizitätskonstanten und Leitfähigkeiten der Mischkörper aus isotropen Substanzen," *Annalen der Physik* **416**, 636–664 (1935).
